# Modelling extreme desiccation tolerance in a marine tardigrade

**DOI:** 10.1038/s41598-018-29824-6

**Published:** 2018-07-31

**Authors:** Thomas L. Sørensen-Hygum, Robyn M. Stuart, Aslak Jørgensen, Nadja Møbjerg

**Affiliations:** 1 Section for Cell Biology and Physiology, Department of Biology, University of Copenhagen, Copenhagen, Denmark; 2 Data Science Laboratory, Department of Mathematical Sciences, University of Copenhagen, Copenhagen, Denmark

## Abstract

It has recently been argued that the enigmatic tardigrades (water bears) will endure until the sun dies, surviving any astrophysical calamities in Earth’s oceans. Yet, our knowledge of stress tolerance among marine tardigrade species is very limited and most investigations revolve around species living in moist habitats on land. Here, we investigate desiccation tolerance in the cosmopolitan marine tidal tardigrade, *Echiniscoides sigismundi*, providing the first thorough analysis on recovery upon desiccation from seawater. We test the influence on survival of desiccation surface, time spent desiccated (up to 1 year) and initial water volume. We propose analysis methods for survival estimates, which can be used as a future platform for evaluating and analysing recovery rates in organisms subjected to extreme stress. Our data reveal that marine tidal tardigrades tolerate extremely rapid and extended periods of desiccation from seawater supporting the argument that these animals are among the toughest organisms on Earth.

## Introduction

Tardigrades are microscopic aquatic animals, widely known for their ability to enter the ametabolic state of cryptobiosis in face of adverse environmental conditions^1–7^. Research into these minute animals and the mechanisms that enable them to enter and exit cryptobiosis has gained considerable international attention, primarily due to the potential translational output related to a thorough understanding of latent life phenomena.

Cryptobiosis can be divided into several sub-categories depending on the environmental cue that induces the state. Specifically, anhydrobiosis refers to cryptobiosis induced by desiccation, cryobiosis to cryptobiosis induced by freezing, and osmobiosis to cryptobiosis induced by elevated external osmotic pressure. Importantly, these three forms of cryptobiosis have in common that the inducing abiotic factors force the organism to deal with excessive water loss. Accordingly, we hypothesise that the molecular and physiological mechanisms underlying the three forms are at least partly overlapping. In addition to the above mentioned forms of cryptobiosis, anoxybiosis, induced by lack of oxygen, was originally suggested by Keilin (1959)^8^, but later disputed by Clegg (2001)^3^, who provided evidence of low levels of metabolism in his best candidate of a true anoxybiote, i.e. embryos of the crustacean, *Artemia franciscana*. The latter finding is problematic if we consider true cryptobiosis to be defined by a complete, but reversible shutdown of metabolism. More recently, we have argued that chemobiosis—a form of cryptobiosis induced by toxicants—may also exist^6,9^.

All tardigrades need a film of surrounding water in order to be in their active, feeding and reproducing stage. However, most of the described species live on land, e.g. in moss-cushions or lichens, where they endure periods of desiccation by entering anhydrobiosis. These species are referred to as semi-terrestrial. The phylum Tardigrada divides into two extant lineages, heterotardigrades and eutardigrades^10–12^. Whereas cryptobiosis has been investigated in a number of semi-terrestrial eutardigrade species^9,13–22^, few studies focus on heterotardigrades, including marine species^9,14,22–24^.

It has recently been argued that tardigrades represent one of the most resilient lifeforms on Earth and that these tiny animals will survive all potential astrophysical catastrophes enduring until the sun dies^25^. Specifically, Sloan and co-workers (2017)^25^ argue that none of the possible cataclysmic events would have the power to boil off all water in the oceans of Earth, and that extremotolerant marine life therefore cannot be annihilated by any astrophysical calamities. The authors erroneously base their analysis on studies performed on tardigrade species living on land— little is actually known of the extremotolerant abilities of marine tardigrades.

**Figure 1:**
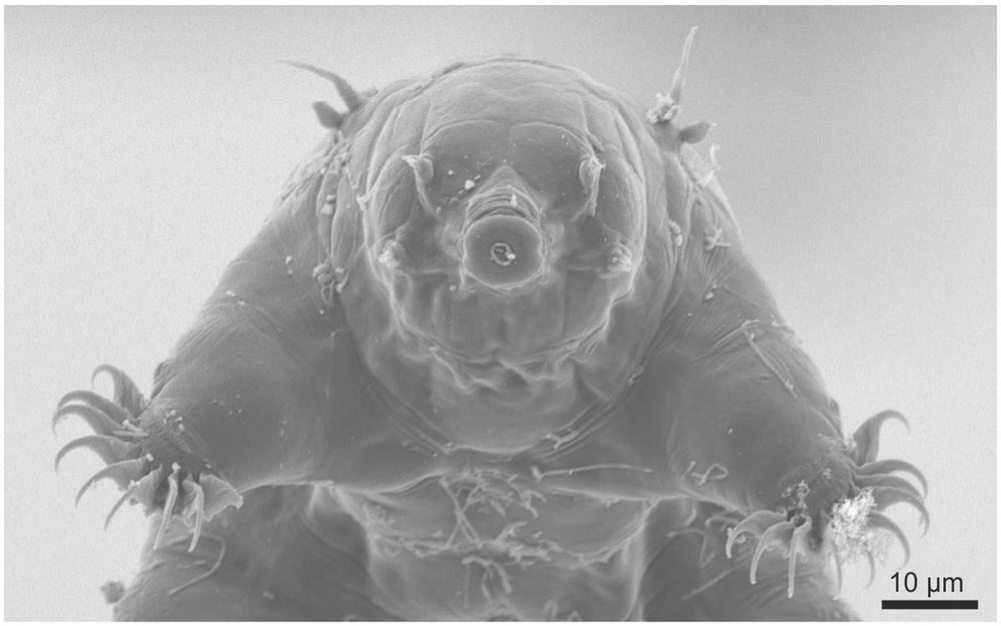
Scanning electron micrograph of active hydrated *Echiniscoides sigismundi* (frontal view).

In the current study, we investigate desiccation tolerance in the marine tidal tardigrade *Echiniscoides sigismundi* (Fig. 1). Our previous investigations provide evidence of a species that is highly tolerant of a range of environmental perturbations, and thus represents a unique model to gain further insights into adaptations to extreme conditions^24,26,27^. *E. sigismundi* is freeze tolerant, it tolerates desiccation from distilled water, as well as from high salinity solutions, and it can undergo cyst-formation^24,26,27^. Here, we provide the first thorough analysis on recovery of *E. sigismundi* following dehydration from seawater, and thus a combined osmobiotic and anhydrobiotic entrance into the quiescent state. The data presented involve an analysis of long-term desiccation (up to 1 year) from natural salinities as well as analyses of the influence of substrate on desiccation tolerance. Based on our data, we: (a) fit generalised linear models (GLMs) to determine the impact of time spent desiccated (taken as a factor) on survival rate for specimens desiccated on filter paper, glass, and hedgehog spines; and (b) estimate a log-logistic model with time spent desiccated (taken as a continuous variable) as the single explanatory variable. Using the GLMs, we estimate the differences in survival rates between different observed desiccation times, and based on the log-logistic model we estimate parameters such as the median lethal desiccation time (i.e., the desiccation time required to achieve 50% mortality). Both the GLMs and the log-logistic model could potentially be employed more generally for evaluating and analysing recovery rates in organisms subjected to extreme stress.

## Results

### Survival of *Echiniscoides sigismundi* preserved in a hydrated and starved condition

**Figure 2:**
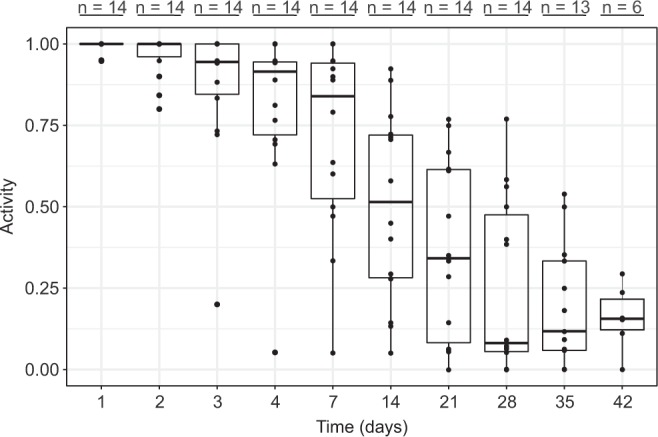
Proportion of active tardigrades (Activity) as a measure of survival in fully hydrated *E. sigismundi*. Tardigrades were kept at 5 °C in filtered seawater without substrate for various time intervals starting from 1 day and up to 42 days. Observed data points (•) are presented with medians (horizontal lines), interquartile ranges (boxes), and 1.5*interquartile ranges (whiskers). Numbers (n) indicate the number of replicate groups.

Control groups were kept hydrated in sterile filtered seawater without substrate for up to 6 weeks (Table 1, Fig. 2). Our data indicate that under the current experimental set-up, fully hydrated *E. sigismundi* can be stored for 2 days without a significant drop in survival, i.e. the distribution of the 1-day and 2-day survival rates were not significantly different (Wilcoxon rank sum test, W = 111, p = 0.47). Accordingly, in desiccation tolerance assays, detection of activity at 2 days after rehydration is likely not affected by the effect of artificial rearing and starvation. Our data further indicate that energy depletion starts to affect the survival of the fully hydrated, active stage *E. sigismundi* after 3 days with a significant drop in mean (standard error) activity from 99% (SE: 1%) after 1 day to 87% (SE: 6%) after 3 days (W = 153, p < 0.01). Activity continues to decline thereafter with a mean activity of 16% (SE: 4%) at t = 6 weeks. The decline in active tardigrades likely reflects energy depletion, but could also represent death by senescence, i.e. 6 weeks is within the life span reported for some tardigrades^28,29^.

**Table 1:** Overview of desiccation experiments on the marine tidal tardigrade *Echiniscoides sigismundi*.

Experiment	Replicate groups*	Total tardigrades**
Controls: no desiccation (kept in seawater)		
	14	**256**
Experiment 1: desiccation on filter paper		
Exp.1, 2 h	9	169
Exp.1, 1 day	8	162
Exp.1, 2 days	5	102
Exp.1, 7 days	6	111
Exp.1, 14 days	6	123
Exp.1, 28 days	11	238
Exp.1, 56 days	6	87
Exp.1, 84 days	6	95
Exp.1, 274 days	5	76
Exp.1, 365 days	6	116
Desiccation on paper total	**68**	**1279**
Experiment 2: desiccation on glass surface		
Exp.2, 0.1 µl, 2 h	7	69
Exp.2, 0.1 µl, 1 day	6	51
Exp.2, 0.1 µl, 2 days	7	68
Exp.2, 0.1 µl, 14 days	6	55
Exp.2, 1 µl, 2 h	4	81
Exp.2, 1 µl, 1 day	6	98
Exp.2, 1 µl, 2 days	9	160
Exp.2, 1 µl, 14 days	3	53
Exp.2, 10 µl, 2 h	6	73
Exp.2, 10 µl, 1 day	6	62
Exp.2, 10 µl, 2 days	4	65
Exp.2, 10 µl, 14 days	3	56
Desiccation on glass total	**67**	**891**
Experiment 3: desiccation on hedgehog spines		
Exp.3, 1 day	5	81
Exp.3, 2 days	7	118
Desiccation on spines total	**12**	**199**
Controls & Experiments total	**161**	**2625**

*Tardigrades were pooled into replicate groups each containing 10–20 specimens.

**Sum of the tardigrades used in all the replicates.

### Desiccation tolerance of *Echiniscoides sigismundi* and the effect of the laying substrates

We assigned tardigrades to experimental series testing desiccation at various time intervals and on different substrates (Table 1). Tardigrades were desiccated on filter paper, a glass surface (hard natured lime glass), and on tips of hedgehog spines, with the latter representing an extremely rapid desiccation time of around 15 seconds. Experiments with desiccation on glass were subdivided into three series with desiccation from different volumes of seawater, i.e. 0.1 µl, 1 µl and 10 µl.

**Figure 3:**
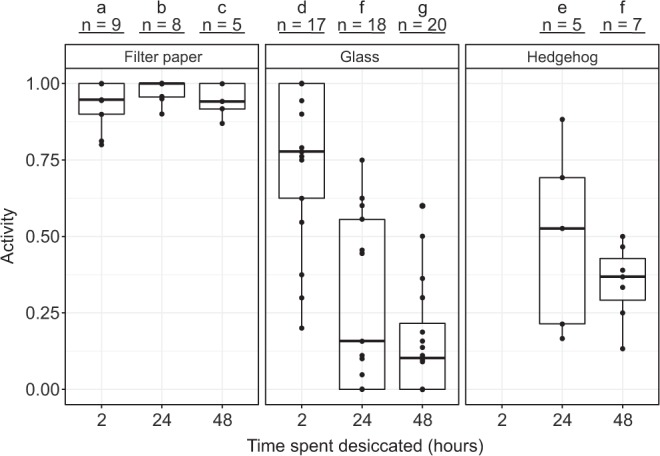
Short-term desiccation of *E. sigismundi* and the effect of laying substrates. Proportion of active tardigrades (Activity) following desiccation on filter paper, glass, and hedgehog spines for periods from 2 to 48 hours. Observed data points (•) are presented with medians (horizontal lines), interquartile ranges (boxes), and 1.5*interquartile ranges (whiskers). Numbers (n) indicate the number of replicate groups. Different letters indicate significant difference at the p ≤ 0.05 level (Tukey’s test).

We found that there was a statistically significant effect of both the desiccation surface (χ^2^(2) = 468.08, p < 0.01) and the desiccation time (χ^2^(2) = 225.93, p < 0.01) on the survival rate of specimens desiccated for up to 48 hours, with specimens desiccated on filter paper having a significantly greater chance of survival than specimens desiccated on glass or hedgehog spines (Fig. 3). This held true regardless of the volume of seawater used; we did not find significant differences in the survival rate when comparing across volumes (χ^2^(1) = 2.162, p = 0.14; Fig. S1), and therefore pooled data for the different volumes in subsequent analyses. We found that a mean of 98% (SE: 1%) of the specimens desiccated on filter paper survived 24 hours of desiccation, compared to 29% (SE: 7%) of the specimens dried on glass (volumes pooled) and 50% (SE: 14%) of the specimens dried on hedgehog spines. Analysing the GLM with Tukey tests revealed that there are significant differences between each of the experimental groups, with the exception of those desiccated on hedgehog spines for 48 hours and those desiccated on glass for 24 hours (Fig. 3).

**Figure 4:**
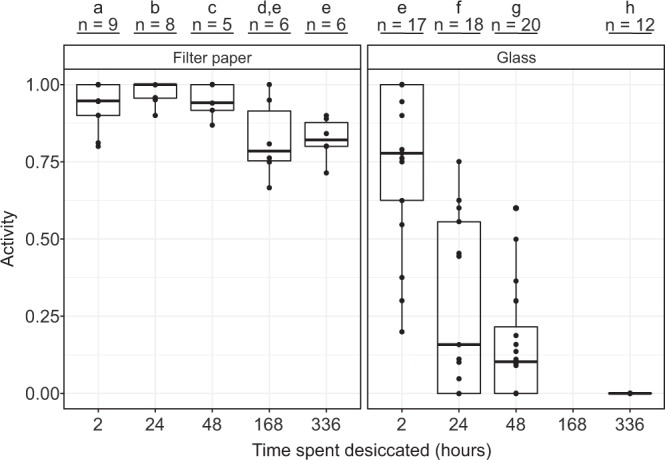
Desiccation of *E. sigismundi* of up to two weeks on filter paper and glass. Proportion of active tardigrades (Activity) following desiccation on filter paper and glass. Observed data points (•) are presented with medians (horizontal lines), interquartile ranges (boxes), and 1.5*interquartile ranges (whiskers). Numbers (n) indicate the number of replicate groups. Different letters indicate significant difference at the p ≤ 0.05 level (Tukey’s test).

The improved survival for specimens desiccated on filter paper compared to glass was also observed for specimens desiccated for up to 2 weeks (Fig. 4), with 0% activity observed in the specimens desiccated on glass after 2 weeks, compared to a mean of 82% (SE: 3%) in the specimens desiccated on filter paper. Based on our GLM, we found a statistically significant effect of both the desiccation surface (χ^2^(1) = 706.03, p < 0.01) and the desiccation time (χ^2^(4) = 362.94, p < 0.01) on the survival rate of the specimens desiccated for up to 2 weeks. Analysing the GLM with Tukey tests revealed that there are significant differences when comparing across desiccation surfaces after any length of time spent desiccated (Fig. 4).

**Figure 5:**
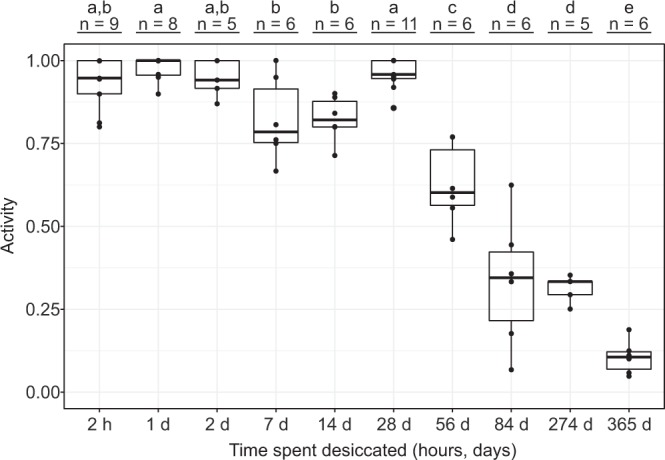
Long-term desiccation of *E. sigismundi*.Proportion of active tardigrades (Activity) following desiccation on filter paper of up to 1 year. Observed data points (•) are presented with medians (horizontal lines), interquartile ranges (boxes), and 1.5*interquartile ranges (whiskers). Numbers (n) indicate the number of replicate groups. Different letters indicate significant difference at the p ≤ 0.05 level (Tukey’s test).

Our results show that filter paper is the optimal desiccation surface and we therefore proceeded using this surface to find a possible upper limit for the time period in which *E. sigismundi* can be preserved in a desiccated state. Compared to the fully hydrated specimens kept without substrate (Fig. 2), *E. sigismundi* desiccated from seawater on filter paper exhibit a significantly expanded life span (Fig. 5). Based on our GLM, we found a statistically significant effect of the desiccation time (χ^2^(9) = 580.77, p < 0.01) on the survival rate of the specimens desiccated for up to 1 year. The data presented in Fig. 5 show no significant difference in activity following periods of desiccation from 2 hours and up to 4 weeks, with a slow decline in activity following 2–3 month in the desiccated state. Some specimens clearly regain activity following a year in the desiccated state, as revealed by a mean activity of 10% (SE: 2%) at t = 365 days. Analysing the GLM with Tukey tests revealed that there are significant drops in the survival rates after 4 weeks, 12 weeks, and 1 year (Fig. 5).

### The toxicity of time: Log-logistic modelling of recovery from cryptobiosis

**Figure 6:**
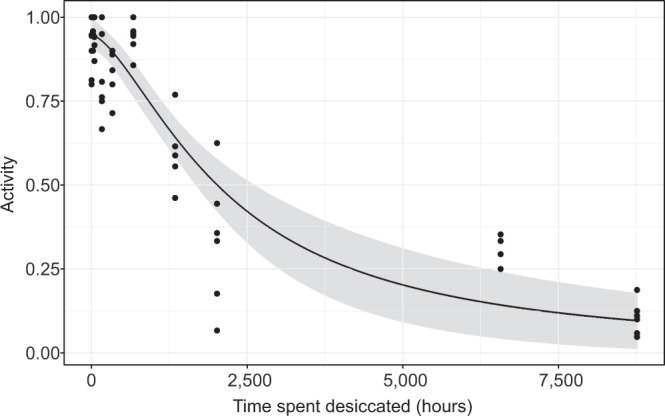
The toxicity of time: Log-logistic model of tardigrade activity (proportion of active tardigrades) following desiccation. Model of tardigrade activity following desiccation on filter paper of up to 1 year. Observed data points (•) are presented together with the model estimate and 95% prediction intervals.

Based on the data of the filter paper desiccated tardigrades we estimated a log-logistic model of survival rate, with time spent desiccated (taken as a continuous variable) as the single explanatory variable. Parameter estimates for $b,d$ and ${LD}50$ are given in Table 2, along with standard errors and significance level. The variance estimate for ${\sigma }^{2}$ was 0.1319. Model predictions, along with confidence intervals, are shown in Fig. 6. The desiccation time required to achieve 50% mortality for *E. sigismundi* under the current conditions is estimated to be 2174 [95% Confidence Interval: 1661–2688] hours, or 12.9 [9.9–16.0] weeks.

**Table 2:** Log-logistic modelling of tardigrade activity following desiccation.

Upper asymptote (${\boldsymbol{d}}$)	0.95 (0.02)***
Slope parameter (${\boldsymbol{b}}$)	1.56 (0.26)***
Inflexion point (${\boldsymbol{LD}}{\boldsymbol{50}}$)	2174 (257)***

***Indicates significance at a 0.1% level.

Parameter estimates for 3-parameter log-logistic model for tardigrade activity, with standard errors provided in brackets (compare to Fig. 6).

## Discussion

A detailed understanding of cryptobiosis comes with an in-depth investigation into selected model organisms combined with a broad comparative knowledge of the phenomenon across species and phyla. Previous studies on desiccation tolerance have focused on various invertebrates, including nematodes, rotifers and the brine shrimp, *Artemia salina*^30–32^. Within tardigrades focus has been on semi-terrestrial species^13–15,17,19,20,22,33,34^, and little is known of desiccation tolerance in marine tardigrades^23,24^. In the current study we investigate desiccation tolerance in the marine tidal cosmopolitan, *Echiniscoides sigismundi*, a species that belongs to the poorly studied heterotardigrade lineage.

Our data reveal that *E. sigismundi* is capable of withstanding extended periods of desiccation with a mean recovery of 10% after a year in the desiccated state (Fig. 5). It would seem that *Echiniscoides* is less tolerant of long-term desiccation, when compared to semi-terrestrial tardigrades. Specifically, the semi-terrestrial eutardigrade, *Ramazzottius oberhaeuseri*, tolerates desiccation for 1192 days with a mean activity recovery of 21.7%, whereas a mean activity of 9.9% was recorded for the semi-terrestrial heterotardigrade, *Echiniscus* spp., following 706 days of desiccation^14^. A similar pattern revealing a somewhat lower tolerance in *Echiniscoides* as compared to semi-terrestrial tardigrades was observed in a study on tolerance to gamma radiation^35^. The latter may simply reflect a variation in the tolerance level of specific tardigrade species, but, importantly, could also support the argument that electrolyte solutions have a detrimental effect on the ability to recover from extreme stress, as previously reported for semi-terrestrial species^36^.

In the current investigation, a significant drop in recovery was observed in *Echiniscoides* following desiccation for more than 8 weeks, indicating that single specimens start dying after 8 to 12 weeks of desiccation (Fig. 5). These specimens may fail to regain activity due to damage of macromolecules and tissues that cannot be repaired while in the dormant state. One could also argue that the tardigrades may not be fully ametabolic and that the decline in activity consequently represents energy-depletion. In any case, desiccation clearly expands the life span of *E. sigismundi* as revealed by a comparison between the activity of controls (Fig. 2) and filter paper desiccated specimens (Fig. 5). Importantly, at the population level, *E. sigismundi* is likely able to survive >1 year of quiescence as only a few survivors would be needed to re-establish the population. As holds for many of the extreme adaptations reported among tardigrades^16,35,37,38^, the time endured in the quiescent state seems as a bit of a paradox, as the tardigrades under natural conditions would not be desiccated for such a long period of time^39^.

Our data on desiccation of *E. sigismundi* on glass indicates that this substrate inflicts a time dependent toxic effect on the tardigrades’ ability to recover, likely due to a direct interaction between the glass and tardigrade tissue, highlighting the importance of optimising the substrate for experiments on desiccation. Specifically, comparing data from the filter paper and glass desiccation experiments we see that 82% of the tardigrades are active following 2 weeks of desiccation on filter paper, whereas 0% are active following the same period on glass surface (Fig. 4). Interestingly, our glass desiccation data reveal that the rate of desiccation clearly is of less importance for survival than the substrate. During experimentation we, nevertheless, observed aggregation behaviour in *E. sigismundi*. As the water evaporated around the tardigrades and the point of complete desiccation approached, tardigrades would seek remaining water often huddling together and dehydrating in smaller groups. This observation is in concordance with experiments on *Richtersius coronifer*, documenting increased survival for aggregated specimens^34^. The latter study on *R. coronifer* did, however, not analyse behaviour, but rather quantified the influence of experimentally forced aggregation on anhydrobiotic survival. In the current study on *E. sigismundi*, aggregation behaviour was observed in all experimental series, except the hedgehog spine series, where such grouping was prevented by the low numbers of tardigrades (i.e. 1–3 specimens) transferred to the spines as well as the extremely fast rate of desiccation. Our results on desiccation from hedgehog spines indicate that extremely rapid desiccation is not immediately fatal to this species. The latter finding is in clear contrast to previous observations on semi-terrestrial species, which suggested that the rate of desiccation is of vast importance for recovery^1,33^. It is likely that this discrepancy is at least partly due to variation in the adaptation profiles of the specific tardigrade species.

It has been suggested that relative humidity is an important factor influencing post-cryptobiotic survival in tardigrades^1,33,40^. Our current analyses as well as previous studies on strong cryptobionts (e.g. *Richtersius coronifer*, *Ramazzottius* spp., *Echiniscoides sigismundi*) seem to contradict this hypothesis^7,19,24,33^. Relative humidity may, nevertheless, be of vast importance for tardigrades and other invertebrates that are less resilient, as for example *Hypsibius dujardini*, a model species currently used for studies on tardigrade development, oogenesis and various genome projects^41–44^.

In summary, we present a thorough analysis of data on desiccation tolerance, with marine tardigrades having spent up to 1 year in the desiccated state. We propose a valid log-logistic model for post-cryptobiotic survival with time spent desiccated as the single explanatory variable. This model as well as the proposed GLMs could potentially be employed more generally for evaluating and analysing recovery rates in organisms subjected to extreme stress.

## Methods

### Tardigrade sampling

Tardigrades, *Echiniscoides sigismundi*, were sampled from barnacles settled on rocks near the shoreline at Lynæs, North Zealand, Denmark (55°56′52.3′′N, 11°51′07.8′′E) in the period January 2013 to February 2015. The water temperature during samplings varied from −0.8 °C to 23.2 °C, while salinity ranged from 17.7‰ to 23.6‰. Barnacle shells with *E. sigismundi* were brought to the laboratory, cleaned of soft tissue and kept at 5 °C in locality seawater for periods of up to 4 weeks.

### Experimental procedure

Highly active, mainly adult tardigrades (no sex determinations were made) were collected from the shells and transferred to watch glasses using a stereo microscope and a Pasteur pipette. Specimens were randomly pooled into groups of 10–20 tardigrades and subsequently used as controls or in experimental series with desiccation at various time intervals on filter paper, glass and hedgehog spines (Table 1). Our previous investigations revealed that *E. sigismundi* tolerates desiccation on filter paper in a laboratory environment with no apparent dependency on relative humidity or temperature^24^. Accordingly, tardigrades were desiccated at ambient laboratory temperature (20–27 °C) and relative humidity (27–62%). The tardigrades were considered fully desiccated after exhibiting an easily visible colour change^24^. The desiccation time, representing the time from transfer of the tardigrades onto a given substrate to their complete desiccation (visualised as a change of colour), was measured for each experimental series.

Desiccations on filter paper (Table 1; Experiment 1) were conducted on small ca. 0.5 cm^2^ pieces of paper placed in watch glasses. Groups of ca. 20 tardigrades were transferred to a piece of paper soaked in filtered locality seawater (0.2 µm sterile filters) and desiccated with a mean desiccation time of 23 min (SE: 1 min). The desiccated osmobiotic/anhydrobiotic tardigrades were subsequently kept at 5 °C on the filter paper in watch glasses for 10 different time intervals spanning from 2 hours up to 1 year (Table 1).

For tardigrades desiccated directly on watch glass (Table 1; Experiment 2), groups of ca. 20 specimens were used, with the exception of 0.1 µl experiments, which only included ca. 10 tardigrades per replicate due to space constraints within this small volume of water. Specifically, the experiments with desiccation on glass were subdivided into three series with desiccation from different volumes of seawater, i.e. 0.1 µl, 1 µl and 10 µl, corresponding to mean desiccation times of, respectively, 2 min (SE: 0.4 min), 8 min (SE: 0.5 min) and 37 min (SE: 2 min). The desiccated tardigrades were subsequently kept in the watch glasses at 5 °C for 4 different time intervals.

For a third experimental series (Table 1; Experiment 3), 1–3 tardigrades, only surrounded by a thin film of seawater, were transferred to and desiccated on the tip of a hedgehog spine (*Erinaceus europaeus*) with an extremely rapid mean desiccation time of 15 seconds (SE: 1 sec). Several spines corresponding to a total of ca. 15 desiccated tardigrades were, subsequently, placed on filter paper in watch glasses at 5 °C for either 1 or 2 days.

Tardigrades were rehydrated with 1.5–2 ml of filtered seawater after their assigned period in the desiccated state and if applicable they were gently removed from filter paper in order to ensure an accurate determination of activity. The activity of the tardigrades was evaluated 48 hours post-rehydration under stereomicroscope at x40–50 magnification. Tardigrades were categorised into two groups: *active* animals exhibiting any kind of movement, and *inactive* animals without visible movement. For each replicate group, the proportion of active tardigrades was calculated as the number of active tardigrades divided by the total number of tardigrades in that group.

Control groups of approximately 20 tardigrades were kept hydrated in sterile filtered seawater without substrate in watch glasses at 5 °C for up to 6 weeks (Table 1). The water of the controls was changed every 2 weeks. At extended time points the total number of tardigrades declined in the control groups, due to loss of tardigrades during water changes as well as death followed by disintegration. The latter likely cause a slight overestimation of activity rates in the control series.

### Data analyses and statistics

The analyses of tardigrade activity were divided into 4 separate strands, as outlined below. Firstly, to analyse the longevity of hydrated specimens, we focus on the control experimental series. We estimate the proportion of active tardigrades across all assessments at each time point. Our data are not normally distributed, so we use non-parametric summary measures (medians and interquartile ranges) in plots to evaluate the distribution of survival rates, but report means and standard errors in the text, since these statistics in themselves do not imply a distributional assumption. In addition, we use GLMs and non-linear regression (as explained in greater detail below).

Secondly, to analyse the survival rate over short (up to 48 hours) periods of desiccation, a GLM (logistic regression with a logit link function) was specified with desiccation surface, the volume of seawater used (only relevant for the specimens desiccated on glass), and the time spent desiccated (2 hours, 24 hours or 48 hours, treated as a factor) as the three independent variables, and the proportion of specimens active 48 hours after rehydrating as the dependent variable. We validated the model and tested the significance of the three independent variables (plus their interactions) using approximate likelihood ratio tests, with p < 0.05 considered statistically significant. The volume of seawater used was not found to be statistically significant, so it was dropped from the analysis and we proceeded with the remaining two factors. Tukey post-hoc tests were conducted to contrast the proportion of active specimens in each group.

Thirdly, to analyse the survival rates over medium (up to 2 weeks) periods of desiccation, we focused on the experimental series desiccated on glass and on filter paper (the hedgehog series did not continue beyond 48 hours). We estimated a GLM (logistic regression with a logit link function) with desiccation surface, the volume of seawater used (only relevant for the specimens desiccated on glass), and the time spent desiccated (treated as a factor) as the three independent variables, and the proportion of specimens active 48 hours after rehydrating as the dependent variable. We validated the model and tested the significance of the independent variables (plus their interactions) using approximate likelihood ratio tests, with p < 0.05 considered statistically significant. As the volume of seawater used was not found to be statistically significant (Fig. S1), it was dropped from the analysis and we proceeded with the remaining two factors and conducted Tukey post-hoc tests as described above.

Finally, to analyse the survival rates over long (up to 1 year) periods of desiccation we conducted two analyses. Firstly, we estimated a GLM (logistic regression with a logit link function) to determine the impact of time spent desiccated (treated as a factor) on survival rate for the specimens desiccated on filter paper; the model was then validated and we tested the significance of the independent variable using approximate likelihood ratio tests, with p < 0.05 considered statistically significant. Secondly, we estimated a log-logistic model of survival rate, with time spent desiccated (taken as a continuous variable) as the single explanatory variable. The log-logistic model expresses the proportion of animals active after 48 hours of rehydration as a parametric function of time, as follows:

$$\Pr opActive{48}_{it}=\frac{d}{1+\exp (b(\mathrm{log}(t)-LD50)}+{e}_{it}$$

where ${{PropActive}48}_{{it}}$ is the proportion of animals in replicate *i* which were desiccated for *t* hours and observed to be active after 48 hours of rehydration, and the parameters *d, b* and *LD*50 are estimated in order to minimise the sum of squared differences between the model predictions and the observations. Here, $d$ is the upper asymptote, *b* is the slope and ${LD}50$ is the inflexion point, which also has the interpretation of representing the median lethal desiccation time, i.e. the desiccation time required to achieve 50% mortality. The residuals ${e}_{{it}}$ are assumed to be normally distributed with zero mean and variance parameter ${\sigma }^{2}$.

All analyses were carried out using R: A language and environment for statistical computing (R Core Team, 2016)^45^, with the ggplot2 package^46^ and the software CorelDraw X8 (Corel corporation) used for graphing. We used the drc package^47^ for estimating the log-logistic model. All of the statistical tools employed for these analyses could potentially be employed more generally for evaluating and analysing recovery rates in organisms subjected to cryptobiosis.

### Code availability

Computer codes have been uploaded to GitHub (https://github.com/robynstuart/tardigrades).

### Data availability

Datasets on tardigrade activity generated and analysed during the current study are available from GitHub (https://github.com/robynstuart/tardigrades).

## Electronic supplementary material

Supplementary Material
