# Supplementary material for: Modelling extreme desiccation tolerance in a marine tardigrade
Source: Sci Rep. 2018 Jul 31;8:11495. doi: 10.1038/s41598-018-29824-6 (PMC6068186; doi:10.1038/s41598-018-29824-6)
Supplement: Supplementary file 1 — Supplementary Material [file 41598_2018_29824_MOESM1_ESM.pdf]

Modelling extreme desiccation tolerance in a marine tardigrade

Thomas L. Sørensen-Hygum[1], Robyn Margaret Stuart[2], Aslak Jørgensen[1], Nadja Møbjerg[1*]

[1]Section for Cell Biology and Physiology, Department of Biology, University of Copenhagen, Denmark

[2]Laboratory for Applied Statistics, Department of Mathematical Sciences, University of Copenhagen, Denmark

*Correspondence should be addressed to N.M. (e-mail: nmobjerg@bio.ku.dk)

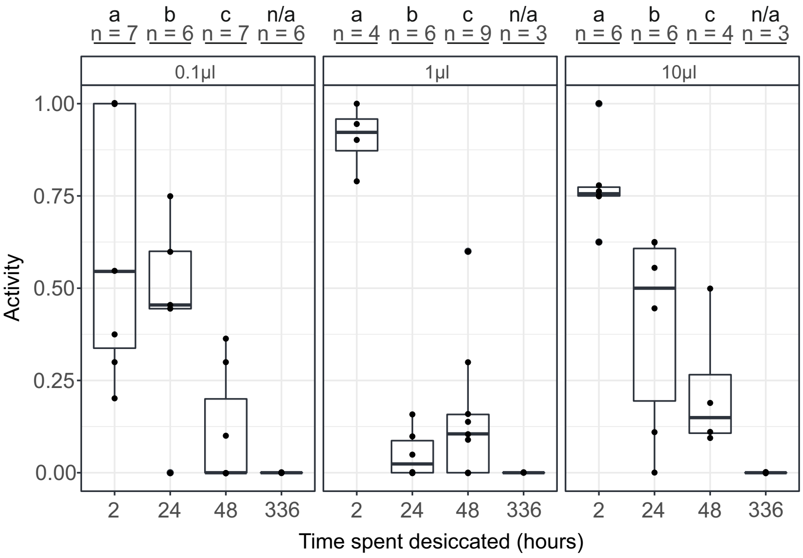

a
n = 7
b
n = 6
c
n = 7
n/a
n = 6
a
n = 4
b
n = 6
c
n = 9
n/a
n = 3
a
n = 6
b
n = 6
c
n = 4
n/a
n = 3
0.1µl
1µl
10µl
Activity
1.00
0.75
0.50
0.25
0.00
2
24
48
336
Time spent desiccated (hours)

Figure S1| Desiccation of *E. sigismundi* on glass from different volumes of seawater. Tardigrade activity following desiccation on hard natured lime glass from different volumes of seawater. Observed data points (•) are presented with medians (horizontal lines), interquartile ranges (boxes), and 1.5*interquartile ranges (whiskers). Numbers (n) indicate the number of replicate groups. Different letters indicate significant difference at the $p \leq 0.05$ level (Tukey's test).
